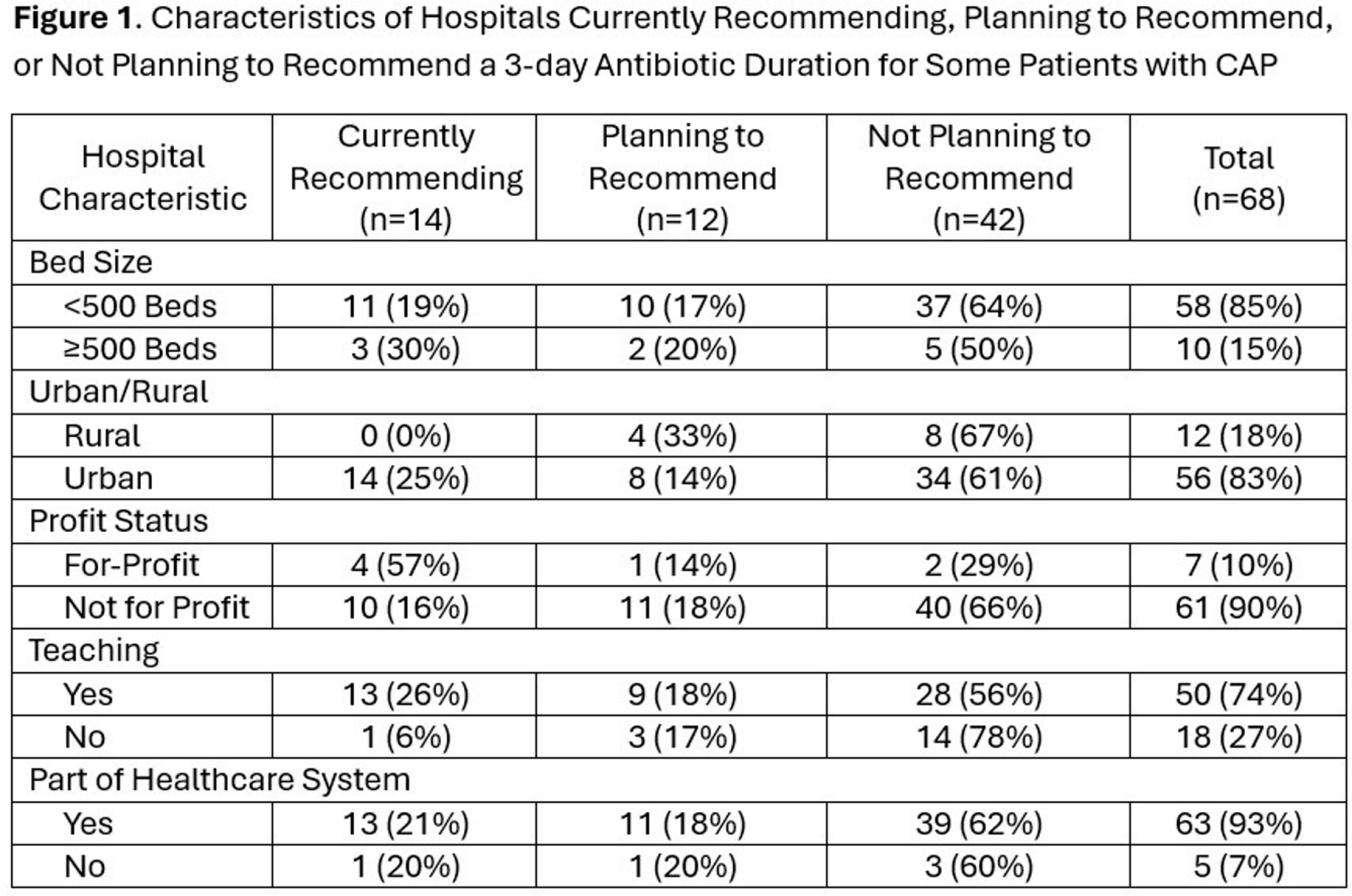# 89 Infection Preventionists as Trained Observers: A Sustainable Model for Safety in High-Level Isolation Care

**DOI:** 10.1017/ash.2026.10514

**Published:** 2026-06-23

**Authors:** Valerie Vaughn, Jennifer Horowitz, Ashwin Gupta, Tejal Gandhi, Lindsay Petty, Paul Bozyk, Megan Cahill, Lisa Dumkow, Rania Esteitie, Anurag Malani, Preeti Misra, Robert Neetz, David Paje, Danielle Osterholzer, Mariam Younas, Andrea White, Tawny Czilok, Elizabeth McLaughlin, Scott Flanders, Julie Szymczak

**Affiliations:** 1 University of Utah School of Medicine; 2 DIvision of Hospital Medicine, Michigan Medicine; 3 University of Michgian/VA Ann Arbor Healthcare System; 4 University of Michigan Medical School; 5 Michigan Medicine; 6 Trinity Health Grand Rapids; 7 Trinity Health Michigan; 8 Trinity Health Livonia Hospital; 9 MyMichigan Health; 10 University of Michigan; 11 Michigan State University College of Human Medicine; 12 Michigan State University - College of Human Medicine, Hurley Medical Center

## Abstract

**Background:** The 2025 American Thoracic Society Community-acquired Pneumonia (CAP) guidelines conditionally recommend that patients hospitalized with non-severe CAP who reach clinical stability be treated with <5 days (3 day minimum) of antibiotics. To determine whether hospitals were implementing or planning to implement this recommendation, we surveyed 68 hospitals participating in the Michigan Hospital Medicine Safety Consortium (HMS). **Methods:** Hospitals participating in HMS completed an electronic survey in October and November of 2025. Abstractors were asked to answer (with stewardship team input) whether their hospital recommended (or planned to recommend) 3-day antibiotic treatment for any patients with CAP. If yes, they were asked a) which patient subgroups, b) whether their hospital guideline recommended 3 days, and c) what stewardship interventions they had implemented to increase 3-day durations. All hospitals were asked about barriers. **Results:** All hospitals (68/68) responded to the survey; 21% (14) reported they currently recommend a 3-day duration for at least some patients with CAP; another 18% (12) do not but are planning to. Hospital characteristics by response category are shown in Figure 1. Of hospitals already recommending or planning to recommend a 3-day duration, 31% (8) recommend it (or plan to) in all patients with CAP. Among the 18 hospitals recommending/planning to recommend a 3-day duration for only a subset of patients, commonly stated criteria were: clinical stability (55%), non-severe CAP (33%), or uncomplicated CAP (33%). Most hospitals (79%) recommending a 3-day duration included the 3-day recommendation in their CAP guideline and 71% reported at least one stewardship intervention in place to increase use of 3-day durations (10, education; 3, audit and feedback; 2, pharmacist bundle; 1, orderset; 1, discharge-specific intervention). Commonly reported barriers or reasons for not recommending a 3-day duration included: focus on 5-day duration (31%), resource/time limitation (14%), EHR transition (12%), and inability to recommend a 3-day duration unless the entire healthcare system did (10%). Notably, 7% expressed confusion about IDSA endorsement of the ATS guidelines (IDSA endorsed the <5 day duration but not the viral pneumonia recommendation). **Conclusion:** Just over a third (38%) of Michigan hospitals currently recommend or plan to recommend a 3-day antibiotic duration for at least some hospitalized patients with CAP. For the remaining 62% of hospitals, continued focus on 5-day duration, standard barriers around stewardship infrastructure, and concerns about existing evidence, safety, and guidance limit enthusiasm for a 3-day duration.

**Figure:**